# Case report: superficial kaposiform hemangioendothelioma without the Kasabach–Merritt phenomenon

**DOI:** 10.3389/fonc.2026.1827436

**Published:** 2026-05-18

**Authors:** Yeli Chen, Ziyu Huang, Feifei Huang, Jianna Chen, Yazhou Wang

**Affiliations:** 1 Department of Dermatology and Aesthetic Plastic Surgery, Hainan Women and Children’s Medical Center, Haikou, China; 2 School of Pediatrics, Hainan Medical University, Haikou, China; 3 Department of Radiology, Hainan Women and Children’s Medical Center, Haikou, China; 4 Department of Pathology, Hainan Women and Children’s Medical Center, Haikou, China; 5 Department of Cardiovascular Disease, Hainan Women and Children’s Medical Center, Haikou, China

**Keywords:** case report, histopathology, kaposiform hemangioendothelioma, magnetic resonance imaging, sirolimus

## Abstract

Kaposiform hemangioendothelioma (KHE) is a rare intermediate-grade malignant vascular tumor that is frequently associated with the Kasabach–Merritt phenomenon (KMP). Cases without KMP are less common and, therefore, prone to misdiagnosis. We report a case of superficial KHE in a 6-month-old male infant without associated KMP. Magnetic resonance imaging revealed a poorly demarcated lesion with T1 hypointensity and T2 hyperintensity, demonstrating marked enhancement and an arterial flow void. The patient was hospitalized because KHE was suspected and received a single course of intralesional pingyangmycin therapy. One week later, histopathological examination showed proliferating spindle-shaped endothelial cells in a glomeruloid pattern. The immunohistochemical results revealed CD31 and CD34 positivity and GLUT1 and D2–40 negativity. GLUT1 negativity helped exclude infantile hemangioma, whereas D2–40 negativity ruled out lymphatic malformation. Upon confirming KHE diagnosis, we initiated a combined sirolimus and prednisone treatment. At the 3-month follow-up, clinical observation showed regression of the lesion, prompting prednisone discontinuation while continuing sirolimus maintenance therapy. In this report, we also discuss the clinical manifestations, imaging features, histopathological characteristics, and treatment options for this KHE subtype and review relevant literature. This case highlights the diagnostic features and therapeutic considerations of superficial KHE without KMP, emphasizing the importance of multidisciplinary evaluation.

## 1 Introduction

Kaposiform hemangioendothelioma (KHE), classified as a locally aggressive vascular neoplasm, has a population-based incidence rate of 0.91/100,000. Approximately 91.8% of patients develop lesions within the first year of life, often accompanied by a life-threatening coagulopathy known as the Kasabach–Merritt phenomenon (KMP) (1). Although numerous reports have described deep or KMP-associated KHE, literature on superficial KHE without KMP remains scarce, increasing the risk of misdiagnosis and delayed treatment, as it can resemble other benign vascular anomalies in infants.

Although biopsy remains the gold standard for diagnosis, imaging—being more readily accepted in pediatric patients—can provide diagnostic clues and help assess tumor extent. Given the heterogeneous nature of KHE, treatment must be individualized, with common approaches being surgery, sirolimus, vincristine, and embolization (2). We present a case of superficial KHE in a pediatric patient and analyze its imaging and histopathological features, as well as therapeutic strategies, to provide insights into the early diagnosis and management of this rare tumor subtype.

## 2 Case description

**Figure 1:**
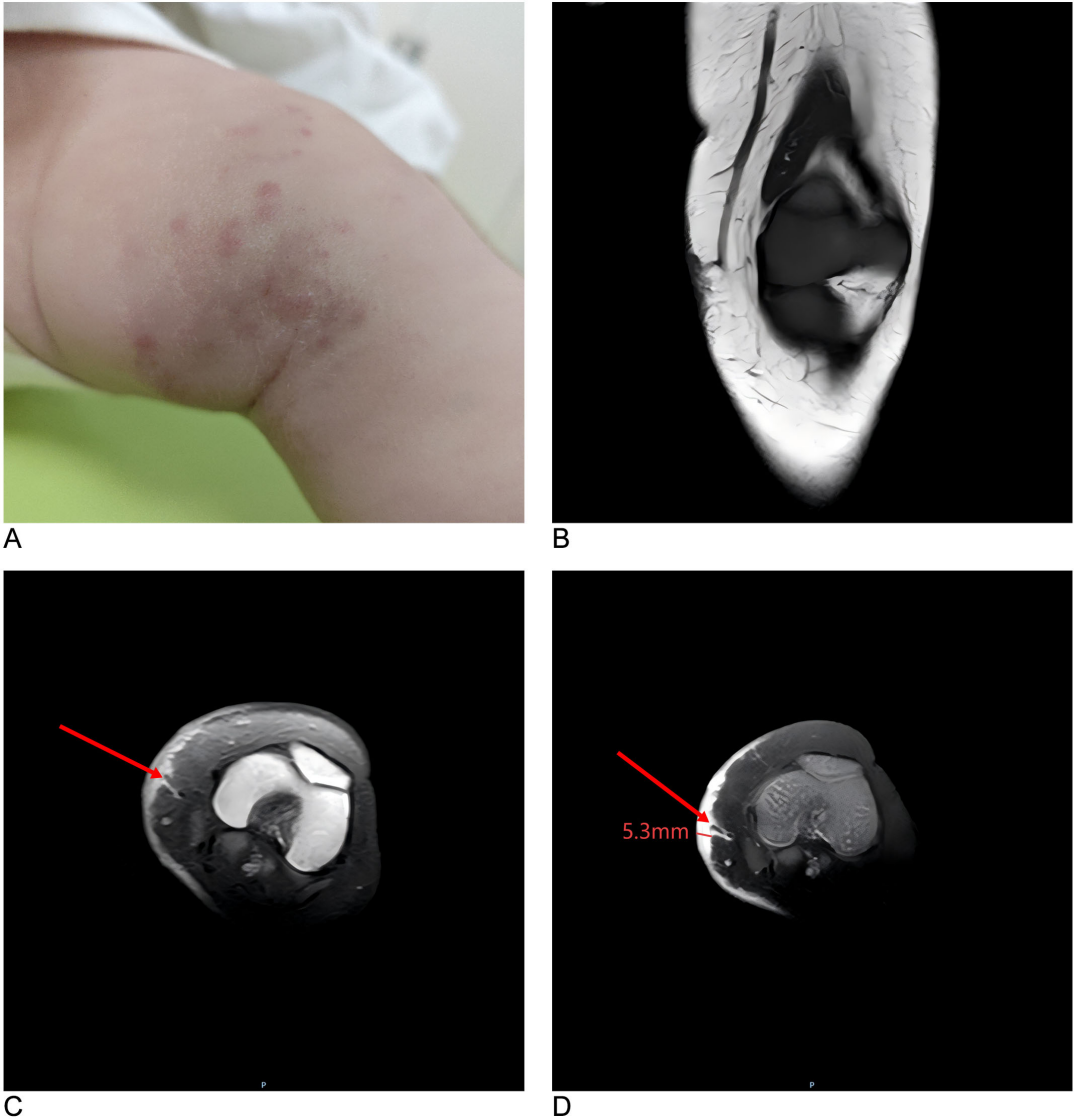
Clinical and MRI examination of the pediatric patient. **(A)** Clinical examination shows a cutaneous mass on the left knee, with multiple purplish-red papules distributed on the surface. **(B, C)** MRI reveals a poorly demarcated, patchy lesion in the medial epidermal and subcutaneous tissues of the left knee, demonstrating hypointensity on T1-weighted coronal image and hyperintensity on T2-weighted axial image. **(D)** On contrast-enhanced T1-weighted axial image, the lesion shows a marked homogeneous enhancement, with abundant vascular supply and drainage. The arrow points at a slightly enlarged arterial flow void. MRI, magnetic resonance images.

A 6-month-old male infant presented with a purplish-red mass on the left knee persisting for 2 months after the development of scattered bluish papules. The patient received topical treatment (unspecified details) at an external hospital but showed no improvement. The lesions progressively enlarged, increased in number, and became redder. Thereafter, the child was transferred to our institution on August 11, 2025. No fever, bleeding, or weight loss was reported during the disease course. No abnormalities were noted in the child’s birth or medical or family histories. Physical examination revealed stable vital signs. A cutaneous mass was observed in the left knee, featuring multiple purplish-red papules distributed over a clinical surface of approximately 70 mm length × 40 mm width. The lesion was firm on palpation, with an infiltrative texture, was immobile, and without ulceration (**Figure 1A**). Laboratory investigations, including complete blood count, prothrombin time, activated partial thromboplastin time, and fibrinogen and D-dimer levels, were within normal ranges. Ultrasonography showed an ill-defined, homogeneous, hyperechoic area (~5 mm thickness) in the subcutaneous tissue of the left knee, with minimal internal blood flow on color Doppler.

Magnetic resonance imaging (MRI) was performed on August 12, using a 3.0T Philips Ingenia scanner with a body coil; a skin biopsy was also performed on the same day. During MRI, the patient rested in a supine position, and after plain scanning, gadodiamide contrast agent (0.2 mmol/kg body weight) was intravenously administered via the antecubital vein at 2 mL/s. The scanning parameters were as follows: T1-weighted imaging: repetition time (TR) 500 ms, echo time (TE) 20 ms; T2-weighted imaging: TR 3119 ms, TE 75 ms; proton density-weighted imaging: TR 2360 ms, TE 25 ms; diffusion-weighted imaging: TR 3775 ms, TE 110 ms, b-values = 0–800 s/mm²; slice thickness 3 mm, slice-gap 0.3 mm, and field of view 200 mm × 220 mm. The scan revealed a lesion involving the medial epidermal and superficial subcutaneous soft tissues of the left knee with ill-defined borders and irregular margins (maximal dimensions of 75.2 mm length × 51.7 mm width × 5.3 mm depth). It exhibited T1 hypointensity and T2 hyperintensity, with marked homogeneous enhancement upon contrast. Multiple small venous structures and a slightly enlarged arterial flow void (~1.8 mm diameter) were observed in the adjacent subcutaneous fat layer (**Figures 1B–D**). No abnormalities were detected in the bones, articular cartilages, menisci, or left knee ligaments.

Based on the MRI findings and the lesion’s location, size, and absence of KMP, we strongly suspected KHE and promptly initiated local pingyangmycin injection therapy on August 13. The treatment solution was prepared by mixing 8 mg pingyangmycin with 0.4 mL dexamethasone (5 mg/mL) and 4 mL iohexol, then diluted with normal saline to 8 mL (final concentration: 1 mg/mL). Then, 2.5 mL of the solution (2.5 mg pingyangmycin, approximately 0.34 mg/kg body weight) was administered via multiple-point injections under digital subtraction angiography guidance, followed by 3 min of manual compression to enhance drug retention. This constituted the sole treatment session. Local (skin erythema, induration, necrosis) and systemic (allergic reactions, pulmonary symptoms) adverse effects were not observed upon closely monitoring this case.

**Figure 2:**
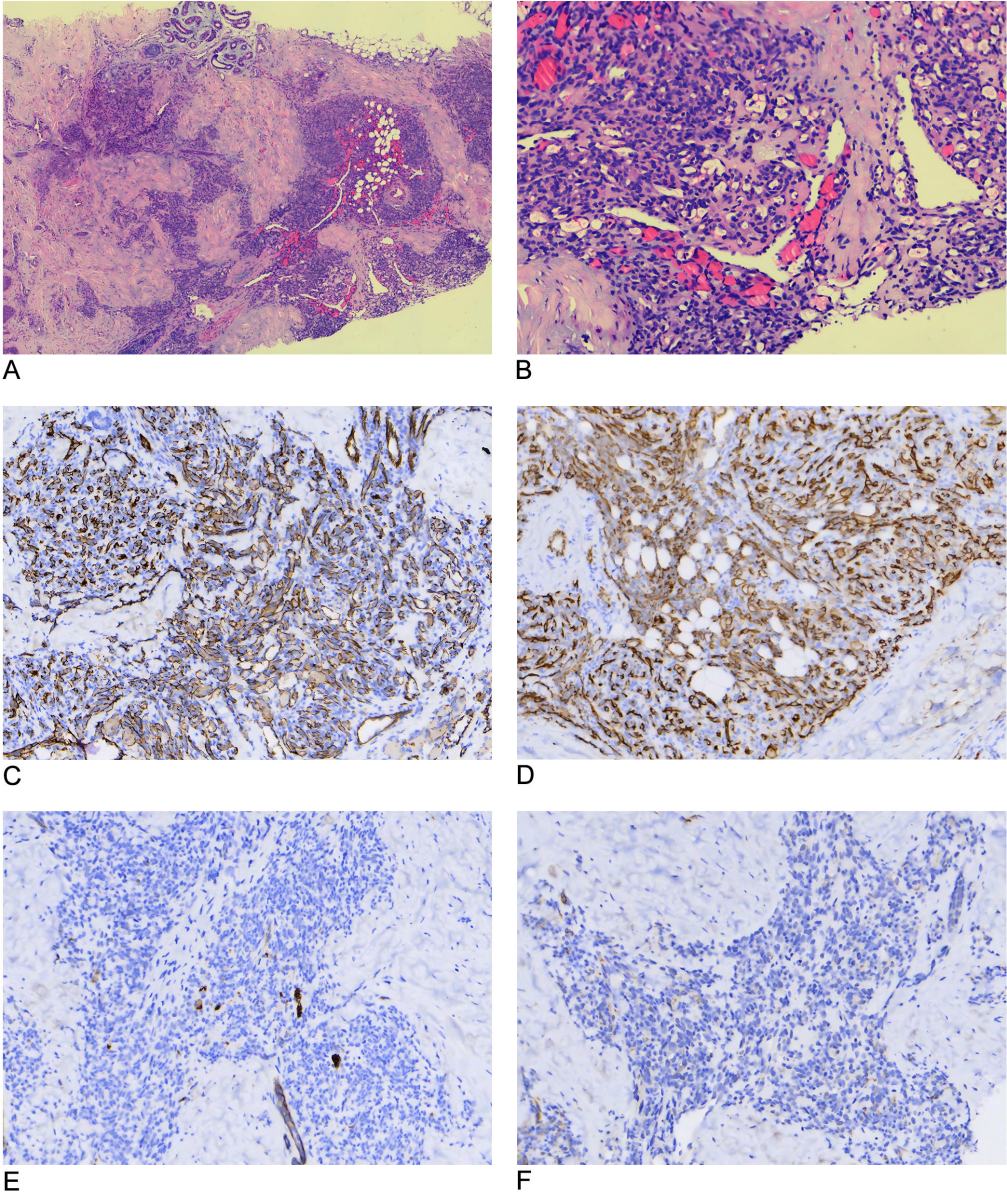
Histopathological and immunohistochemical features of kaposiform hemangioendothelioma. **(A)** The tumor is located in the subcutaneous tissue and composed of multiple hemangioma-like nodules (H&E, × 40). **(B)** Proliferation of spindle endothelial cells in fascicular patterns along with glomeruloid structures and slit-like vascular channels containing erythrocytes (H&E, × 100). **(C, D)** CD31 and CD34: diffusely positive (IHC, × 100). **(E, F)** GLUT1 and D2-40: negative (IHC, × 100). H&E, hematoxylin and eosin; IHC, immunohistochemistry.

After MRI, a skin biopsy specimen was obtained from the red papule on the lesion surface, fixed in 3.7% neutral formaldehyde solution, dehydrated, and paraffin-embedded. Sections were cut at approximately 4 μm thickness and stained using the Envision method. The antibodies used were CD31 (Zhongshan Golden Bridge Biotechnology, Beijing; control: hemangioma), CD34 and glucose transporter protein 1 (GLUT1) (Maixin Biotechnology Development, Fuzhou; controls: hemangioma and placenta, respectively), and D2-40 (GeneTech, Shanghai, Company Limited; control: mesothelioma). All staining procedures were performed using a fully automated immunohistochemical staining system. The results were reported after 1 week, showing fascicular proliferation of short, spindle-shaped vascular endothelial cells with slit-like luminal spaces and a glomeruloid structure. Immunohistochemical analysis revealed that the cells were strongly positive for the vascular endothelial markers CD31 and CD34 and negative for GLUT1 and the lymphatic endothelium marker D2-40 (**Figure 2**). Owing to the unavailability of prospero homeobox-1 (PROX-1) and herpes virus-8 (HHV-8) immunohistochemical markers in our laboratory, these tests could not be conducted.

Consequently, the patient was diagnosed with KHE. We initiated oral medication on September 19: sirolimus at an initial dose of 0.3 mL per administration (8 kg body weight, 66 cm height, equivalent to 0.75 mg/m²) administered twice daily, with a target trough concentration of 5–10 ng/mL. This treatment was planned for a minimum course of 12 months. Concurrently, oral prednisone (2 mg/kg/day) was administered for 1 month, followed by gradual tapering and discontinuation within 3 months. During treatment, trough levels of sirolimus were monitored as scheduled (requiring fasting before blood draw on the morning of testing, before medication administration). At 3 weeks, the first trough level was 1.42 ng/mL, below the target range. Thus, the dose was adjusted to 0.4 mL per administration (1 mg/m²) twice daily. Subsequent monthly monitoring revealed trough levels of 6.73 ng/mL on November 14 (dose unchanged) and 8.66 ng/mL on December 15. After two consecutive measurements within the target range (5–10 ng/mL), monitoring was extended to every 3 months. Routine laboratory monitoring included complete blood count, liver and kidney function tests, and coagulation profiles, all of which remained within normal ranges. During treatment, administration of live attenuated vaccines was avoided. No prophylactic antimicrobials were used, and no opportunistic infections occurred during follow-up (contingency plan: sulfamethoxazole would be initiated as prophylaxis if infections occurred during therapy).

**Figure 3:**
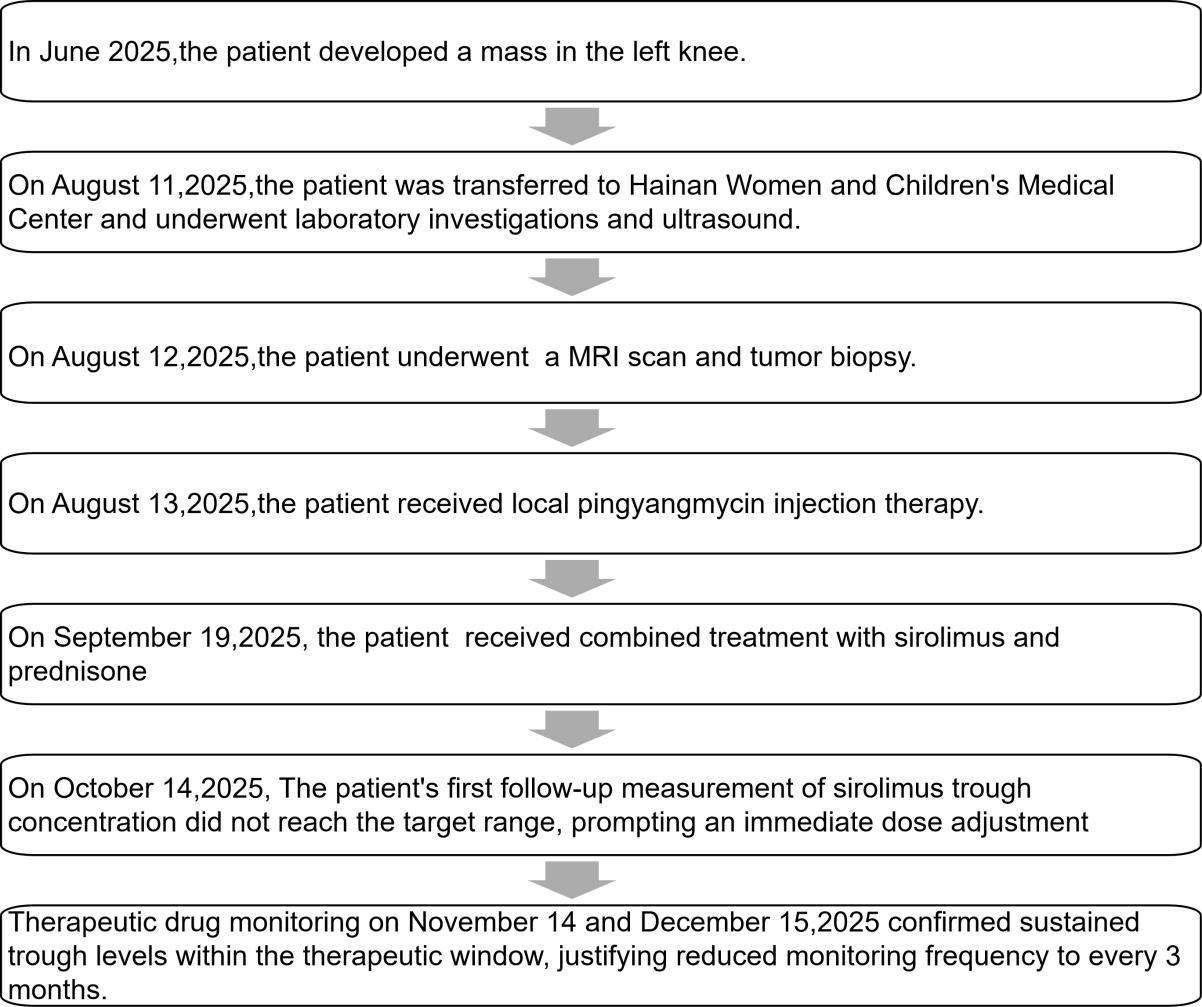
Timeline of the case.

At the 3-month follow-up on November 14, clinical observation showed that the lesion had become lighter in color and softer, and the surface papules had shrunk compared to those pre-treatment. The patient’s family reported that he was well and had no adverse effects such as infection, myelosuppression, or gastrointestinal complications. Consequently, prednisone was discontinued while sirolimus maintenance therapy and monitoring were continued. The timeline of patient’s symptoms, imaging studies, biopsy, treatment, and trough concentration monitoring has been summarized in **Figure 3**.

## 3 Discussion

KHE is a vascular neoplasm with local invasiveness, frequently involving pediatric extremities (thigh predominance), manifesting as an infiltrative, hard, and ill-defined subcutaneous mass with variable red-to-purple discoloration that progressively enlarges without spontaneous regression. In 2013, Croteau et al. (3) first classified KHE into two clinical subtypes based on tumor infiltration depth determined via imaging: superficial (confined to the dermis, subcutaneous tissue, and deep fascia) and deep (located in the mediastinum, retroperitoneum, viscera, or musculoskeletal regions). This classification was later expanded in 2018 by Ji et al. (1), who added a third mixed type (cutaneous involvement with deep infiltration into the muscle, bone, thorax, or retroperitoneum).

Notably, 40–71% of KHE cases are complicated by KMP, characterized by rapid tumor growth, thrombocytopenia, and consumptive coagulopathy (2). Based on findings from major cohort studies, age at onset (<12 months, especially <6 months), tumor maximum diameter (>50 mm, especially >80 mm), and infiltration depth are independent risk factors for KMP. Compared to superficial KHE, mixed-type KHE demonstrates a 2.4–6.3-fold higher likelihood of developing KMP, while deep-type KHE shows a 4–18-fold increased risk, potentially attributable to impaired platelet sequestration due to smaller tumor size, limiting the induction of thrombocytopenia (1, 3–5). Superficial KHE without KMP often leads to delayed treatment due to misclassification as an infantile hemangioma or other vascular anomalies. However, to date, only 150 cases (7–13%) of this subtype have been explicitly reported in English literature, with two case reports (chest/forearm) providing complete imaging, treatment, and follow-up outcomes (6, 7). The present case further supplements the diagnostic and therapeutic data for this subtype occurring in articular regions, providing evidence for early identification.

According to previously reported ultrasound findings in pediatric KHE (8–10), lesions typically appear as ill-defined, hypo- to isoechoic areas with rich vascularity. When infiltrating deep tissues, enhanced echogenicity with a “tree root-like” branching pattern may emerge, representing characteristic changes of invasive growth. In this pediatric case, the superficial lesion presented as an ill-defined hyperechoic area with low vascular signals, allowing the initial exclusion of lymphatic (typically cystic and anechoic) and venous (hypoechoic) malformations (11). However, they lack the characteristic sonographic features of KHE and are difficult to distinguish from other vascular tumors (12, 13). Therefore, further imaging and histopathological examinations are essential for a definitive diagnosis, accurate assessment of lesion extent, and treatment guidance.

MRI is the modality of choice for evaluating KHE as it can assess tumor infiltration depth and differentiate it from other vascular anomalies. When deep tissue invasion or mixed-type KHE is present, complications such as KMP, bone destruction, pericardial effusion, intestinal obstruction, hydronephrosis, or obstructive jaundice may occur. On T1-weighted sequences, the lesions appear as ill-defined masses with isointense or hypointense signals, showing marked homogeneous or heterogeneous enhancement after gadolinium injection. T2-weighted sequences typically demonstrate hyperintense signals, sometimes accompanied by punctate or linear hypointensities corresponding to intralesional hemorrhage or hemosiderin deposition, which are characteristic features of KHE (9, 10). The MRI findings from Peng et al.’s (14) cohort of 64 KHE cases revealed that 14% exhibited dilated, high-flow vascular channels, 43.8% showed infiltrative soft-tissue edema, and 42.2% demonstrated adjacent bone destruction or remodeling. This invasive growth pattern is a key diagnostic feature for distinguishing KHE from other benign vascular anomalies (11, 13). Liang et al. (15) found that osseous KHE exhibited distinct features compared with those of other organs, demonstrating bone destruction with sclerosis and potentially absent post-contrast enhancement. The MRI findings in this case were consistent with the reports described above and showed the characteristic imaging features of KHE.

Histopathology and immunohistochemistry are the gold standards for diagnosing and differentiating KHE. The tumor is composed of small blood vessels and short, spindle-shaped endothelial cells densely arranged in irregular, infiltrative lobules or nodules. These structures exhibit a characteristic glomeruloid pattern and slit-like vascular channels containing variable numbers of erythrocytes, with occasional scattered multinucleated giant cells (16). These features help distinguish KHE from lymphatic and venous malformations, which demonstrate different vascular architectures (17, 18). Immunohistochemically, KHE shows diffuse positivity for CD31 and CD34 and negativity for GLUT1 and HHV-8. Partial positivity may be observed for D2–40 and PROX-1 (2, 16).

GLUT1 and HHV-8 negativity help distinguish KHE from infantile hemangioma and Kaposi’s sarcoma, respectively (16). In studies by Debelenko et al. (19–21), D2–40 and PROX-1 were identified as diagnostic markers (along with GLUT1) for differentiating KHE from other benign infantile vascular tumors; however, some cases (including the present case) demonstrated D2–40 negativity, with expression limited to peripheral lymphatic vessels rather than to tumor cells (7, 22). This may be associated with the following factors: 1) superficial KHE may exhibit lower degrees of lymphatic endothelial differentiation, 2) sampling site variations may influence immunohistochemical results, and 3) tumor heterogeneity may lead to regional expression disparities. Consequently, the definitive diagnosis of KHE necessitates a multidisciplinary approach integrating clinical, imaging, and pathological findings. **Table 1** summarizes the key features that distinguish KHE from its major disease mimickers.

**Table 1:** Key diagnostic features differentiating superficial KHE from other common vascular anomalies in infants.

Entity	Clinical manifestations	Ultrasound	MRI	Histopathology and immunohistochemistry
Superficial KHE	Infiltrative firm subcutaneous mass with ill-defined borders and red-purple discoloration, with or without pain.	An ill-defined solid mass with heterogeneous hypo- to isoechoic echogenicity is observed, which may become more echogenic when infiltrating deep tissues, forming a “root-like” pattern. The lesion exhibits rich vascularity.	T1: iso-/hypointense with marked homogeneous/heterogeneous enhancement T2: hyperintense, possibly accompanied by punctate or linear hypointensities. Ill-defined borders Flow voids Infiltrative growth may lead to soft tissue edema	Fascicular proliferation of short spindled endothelial cells with hyalinized stroma, slit-like vessels, and glomeruloid structures. IHC: CD31/CD34+ (diffusely), D2-40/PROX-1+ (focally), GLUT1/HHV8–.
Infantile hemangioma	Soft, well-circumscribed red-purple mass with growth/regression phases.	A solid mass predominantly involving the epidermis and dermis demonstrates variable echogenicity (hyperechoic/hypoechoic/mixed), well-defined borders, and rich vascularity.	T1: isointense signal with homogeneous enhancement T2: hyperintense signal with well-defined margins Flow voids Fatty signal changes are observed during the involution phase	Lobular and sheet-like dense proliferation of capillaries within the dermis, with plump endothelial cells. IHC: GLUT1/CD31/CD34+, D2-40–
Congenital hemangioma	Purple mass with telangiectasia, often surrounded by a pale halo, and lacks a proliferative phase.	A well-defined heterogeneous mass with vascular anomalies (dilated vessels/venous lakes), dystrophic calcifications, and perilesional edema.	T1: isointense T2: hyperintense with marked enhancement, showing prominent “flow voids” and possible calcifications/thrombosis	Congested capillaries form lobular structures separated by fibrous septa. IHC: CD31/CD34+; GLUT1/D2-40–
Lymphatic malformation	Fluid-filled vesicles or massive tumors.	A cystic anechoic area without apparent blood flow signals, with visible fluid-fluid levels (indicating hemorrhage).	T1: hypointense T2: hyperintense with possible signal heterogeneity due to hemorrhage, showing no central enhancement Fluid levels or debris may be seen within the cyst Microcystic types demonstrate marked enhancement, while macrocystic types exhibit only wall/septal enhancement	Composed of dilated lymphatic channels with variably thickened walls depending on subtype, potentially accompanied by smooth muscle and lymphocyte infiltration. IHC: D2-40/CD31+, CD34–
Venous malformation	A blue or bluish-purple, soft, compressible, non-pulsatile mass that changes with body position, with some cases exhibiting palpable phleboliths.	A well-defined heterogeneous hypoechoic lesion, with some areas showing hyperechoic foci accompanied by posterior acoustic shadowing (phleboliths), demonstrating slow or absent blood flow.	T1: iso-/hypointense T2: hyperintense with flow voids, absence of solid tumor components, demonstrating venous-phase enhancement post-contrast Some cases exhibit round hypointense foci (phleboliths), thrombosis, and fluid-fluid levels	Composed of dilated thin-walled venous vessels with diffuse or localized growth patterns. IHC: CD31/CD34+, GLUT1–

IHC, immunohistochemistry; KHE, Kaposiform hemangioendothelioma; MRI, magnetic resonance imaging

KHE management emphasizes early intervention and individualized approaches to control tumor growth, prevent complications, and minimize sequelae. Observation is reserved for tiny, stable superficial KHE. Despite shallow infiltration and no initial KMP, this case (age at onset <6 months, 75.2 mm tumor diameter) had high KMP progression risk (48% develop secondary KMP 4–6 weeks after diagnosis), precluding observation (1–3). Surgery is the definitive treatment for completely resectable isolated lesions. However, this option was not prioritized owing to lesion size, joint location, and age—factors that confer significant risks of surgical trauma and function impairment. Vincristine was previously considered first-line therapy for KHE with KMP, but its use is now limited to cases in which sirolimus fails or is contraindicated, given its unstable efficacy, slow onset of action, intravenous administration requirements, and neurotoxicity risks (2).

Sirolimus, a mammalian target of rapamycin inhibitor, has recently become an essential therapeutic agent for KHE because it effectively reduces tumor volume and improves hematological abnormalities through its anti-angiogenic effects. Ji et al. (23) conducted a multicenter retrospective study of 52 patients with advanced KHE and demonstrated significant symptom improvement in 96% and 98% of patients at 6 and 12 months of treatment, respectively. Zhou et al. (24) reported a median treatment duration of 21.6 months in 167 patients with KHE, with long-term follow-up (median, 56 months) revealing durable responses in 92.2% of the cases. However, tumor rebound occurred in 17.3% of the patients after therapy discontinuation, indicating the need for adequate treatment duration. Shan et al. (25) demonstrated that sirolimus achieved optimal efficacy when trough levels were maintained at 10–15 ng/mL, with no additional therapeutic benefit observed at concentrations exceeding 15 ng/mL. A randomized controlled trial (26) further confirmed that a low-dose regimen (trough concentration 5–8 ng/mL) demonstrated non-inferior efficacy to higher doses for treating KHE without KMP with fewer adverse effects, providing the rationale for the target trough concentration in this case.

Topical sirolimus (0.1% ointment, twice daily) treatment clinically improves superficial KHE after 6 months; however, radiographic evidence is lacking (27). The aforementioned case report employed a combination of oral propranolol and topical sirolimus for 1 year to treat superficial KHE on the chest, resulting in a reduction of lesion thickness from 6 mm to 2.1 mm (7). Given the lesion’s thickness of 5.3 mm in the present case, topical application could not ensure adequate intralesional drug concentration and was, therefore, not considered for first-line treatment. Compared to sirolimus monotherapy, combination therapy with a short course of prednisone (discontinued within 3 months) reduces secondary KMP risk (28). Pingyangmycin exerts direct pro-apoptotic effects on endothelial cells via intralesional injection, demonstrating synergistic complementarity with systemic therapy. This approach is suitable for large superficial lesions in functional anatomical areas while minimizing systemic drug exposure (29, 30). Owing to these cumulative considerations, the patient achieved initial therapeutic efficacy through this triple therapy.

Long-term follow-up studies (24, 31, 32) revealed that KHE may lead to varying degrees of sequelae, including lymphedema, restricted mobility, growth impairment, and chronic pain. Although superficial KHE generally has a favorable prognosis, it may still result in residual cutaneous lesions: pseudo-port-wine stains with papules, telangiectasia with swelling, and fibrotic subcutaneous infiltrates (3). Therefore, we recommend the following measures: 1) evaluate KMP risk factors upon KHE confirmation to enable prompt treatment initiation; 2) maintain sufficiently prolonged sirolimus therapy (recommended ≥12 months), as premature discontinuation may cause recurrence; 3) conduct regular monitoring of complete blood counts, coagulation profiles, liver/kidney function, and drug trough levels every 3 months, and MRI every 6 months during the maintenance phase. Rapid increases in tumor size/thickness; hardening; new-onset pain; color change; and persistent declines in D-dimer, fibrinogen, and platelet levels should prompt immediate evaluation for KMP; and 4) ensure post-treatment follow-up for at least 2 years to monitor potential recurrence.

This case report systematically describes the diagnosis and treatment of superficial KHE without KMP in the knee joint region, confirming its characteristic imaging and pathological features while exploring the discordance with most previously reported D2–40 positivity cases. Compared with two similar cases treated with oral propranolol plus topical sirolimus or topical tacrolimus with compression therapy (6, 7), our triple-combination therapy demonstrates stronger evidence-based rationale, providing a rapid and standardized therapeutic strategy for superficial KHE in functional anatomical areas. However, this single-case study had inherent methodological constraints, necessitating larger cohorts and extended follow-up to achieve conclusive verification.

## 4 Patient perspective

The patient’s caregivers reported good treatment adherence and satisfaction with clinical improvement.

## Data Availability

The original contributions presented in the study are included in the article/**Supplementary Material**. Further inquiries can be directed to the corresponding author.
